# Long Noncoding RNAs Involved in the Endocrine Therapy Resistance of Breast Cancer

**DOI:** 10.3390/cancers12061424

**Published:** 2020-05-31

**Authors:** Toshihiko Takeiwa, Kazuhiro Ikeda, Yuichi Mitobe, Kuniko Horie-Inoue, Satoshi Inoue

**Affiliations:** 1 Division of Gene Regulation and Signal Transduction, Research Center for Genomic Medicine, Saitama Medical University, Hidaka, Saitama 350-1241, Japan; ttakeiwa@saitama-med.ac.jp (T.T.); ikeda@saitama-med.ac.jp (K.I.); ymitobe31@gmail.com (Y.M.); khorie07@saitama-med.ac.jp (K.H.-I.); 2 Department of Systems Aging Science and Medicine, Tokyo Metropolitan Institute of Gerontology, Itabashi-ku, Tokyo 173-0015, Japan

**Keywords:** breast cancer, long noncoding RNA, endocrine therapy, endocrine therapy resistance

## Abstract

Long noncoding RNAs (lncRNAs) are defined as RNAs longer than 200 nucleotides that do not encode proteins. Recent studies have demonstrated that numerous lncRNAs are expressed in humans and play key roles in the development of various types of cancers. Intriguingly, some lncRNAs have been demonstrated to be involved in endocrine therapy resistance for breast cancer through their own mechanisms, suggesting that lncRNAs could be promising new biomarkers and therapeutic targets of breast cancer. Here, we summarize the functions and mechanisms of lncRNAs related to the endocrine therapy resistance of breast cancer.

## 1. Introduction

For women worldwide, breast cancer is the most common cancer, and one in eight to ten women will develop breast cancer during their lifetime [1,2]. Although the endocrine therapies that target sex hormone receptor signaling pathways are effective treatment for breast cancer, therapy resistance and cancer recurrence remain important issues, and novel therapeutic strategies are required. Recent transcriptome analyses have revealed that a large number of long noncoding RNAs (lncRNAs), which are RNAs that are longer than 200 nucleotides in length and do not encode proteins, are expressed in humans [3,4,5]. LncRNAs play key roles in various biological process and diseases, including cancers [6,7,8,9,10]. In breast cancer, some lncRNAs exert oncogenic or tumor-suppressive functions to control breast cancer pathophysiology, such as invasion and metastasis, and drug resistance; these findings are summarized in a recent review article [11]. In terms of endocrine therapy, selective estrogen receptor modulators (SERMs), selective estrogen receptor degraders or downregulators (SERDs), and aromatase inhibitors, are mainly used as drugs to suppress estrogen signaling. For an experimental model of aromatase inhibitor-resistant breast cancer, cells that can obtain the ability to grow under long-term estrogen deprivation (LTED) conditions are preferentially used. Here, we extensively focus on endocrine therapy resistance-associated lncRNAs in breast cancer by including these drugs and experimental models, and describe the recent findings on their functions and mechanisms.

## 2. Breast Cancer

Breast cancer is the most commonly diagnosed cancer and the leading cause of cancer death in women worldwide [1,2]. According to the *GLOBOCAN 2018* database of the International Agency for Research on Cancer, which estimates the incidence and mortality of several cancers, the number of new cases of breast cancer in 2018 was estimated at 2,088,849, and those of deaths due to breast cancer are estimated as 1,276,106 [2]. Breast cancer is classified into at least four subtypes (luminal A, luminal B, human epidermal growth factor receptor 2 (HER2)/erythroblastic oncogene B 2 (ErbB2)-enriched, and basal-like) based on gene expression patterns [1,12,13]. The luminal subtypes are sex hormone receptor-positive [estrogen receptor (ER) or progesterone receptor (PR)-positive] and HER2-negative, and the HER2-enriched subtype is HER2-positive, while the basal-like subtype is ER-, PR-, and HER2-negative. The majority of breast cancers belong to luminal subtypes and are primarily sensitive to estrogen and progesterone [14,15,16]. The receptors of these hormones, ER and PR, respectively, function as ligand-dependent transcription factors. After binding to their ligands, these hormone receptors dimerize and associate with DNA through their DNA-binding domains. These hormone receptors form complexes with other transcription factors and co-regulators, such as the steroid receptor coactivator (SRC)/p160 family proteins and CREB-binding protein (CBP)/p300, and control the transcription of their target genes [17,18,19]. As sex hormone signaling pathways are essential for breast cancer pathophysiology, therapies targeting the hormones and their receptors, or endocrine therapies, remain the standard treatment for breast cancer [20,21]. For instance, drugs that suppress estrogen signaling or estrogen production are used for endocrine therapies. To suppress estrogen-mediated ER activation, drugs such as SERMs and SERDs are used. Although both SERMs and SERDs compete with estrogen, their mechanisms for the regulation of ER signaling are different. SERMs affect the interaction between the ER and co-factors, leading to changes in ER-targeted gene expression. Thus, SERMs, such as tamoxifen and raloxifene, act as ER antagonists in breast cancer and are used for breast cancer therapy or prevention. In contrast, SERDs mediate the destabilization of the ER to abolish ER signaling [21]. In addition to these modulators of the ER, drugs that block estrogen synthesis, such as aromatase inhibitors and luteinizing hormone-releasing agonists, are used for breast cancer treatment [20]. Although endocrine therapies are initially successful, breast cancers eventually acquire resistance to these therapies [22,23]. Moreover, patients with basal-like or triple-negative breast cancer (TNBC) exhibit poor outcomes, because this subtype lacks the expression of ER, PR, and HER2, and its effective therapeutic targets remain unidentified. Furthermore, metastatic breast cancer is considered incurable with the therapies available currently [1,24]. Thus, novel therapeutic targets and biomarkers are urgently needed. Recent studies have shown that lncRNAs play important roles in the pathophysiology of various cancers, including breast cancer, suggesting the potential of lncRNAs in developing novel strategies of cancer treatment [9,10].

## 3. LncRNAs

Together with the advancement of technologies of cDNA cloning and RNA sequencing, ~70–90% of mammalian genomes are shown to be transcribed to produce huge numbers of noncoding RNAs (ncRNAs), while less than 3% of these genomes are translated to proteins, suggesting the importance of ncRNAs in biological processes [25,26,27]. ncRNAs are classified by their length, i.e., ncRNAs shorter than 200 nucleotides are classified as small ncRNAs, while longer ncRNAs are defined as lncRNAs. MicroRNAs (miRNAs) belong to the small ncRNA category and are involved in translational repression and mRNA destabilization in cooperation with various proteins, including argonaute (AGO) proteins [28]. As it has been shown that miRNAs play key roles in numerous biological processes and diseases, including various types of cancers, their clinical application has been studied [10,29]. Moreover, lncRNAs have been suggested to be essential for cell physiology. Previous studies have identified a large number of lncRNA genes in mammals. For example, the GENCODE project, which is part of the ENCODE project and aims to annotate all gene features in the mouse and human genomes, has identified 13,197 and 17,952 lncRNA genes in mice and humans, respectively [30]. Moreover, a previous transcriptome study reported 58,648 lncRNA genes in humans [5]. Although most lncRNAs remain to be studied, it has been gradually elucidated that some lncRNAs play important roles in multiple biological phenomena, such as cell differentiation and organogenesis and diseases [6,7,8]. The expression of lncRNAs tends to be highly cell type- and tissue-specific [3], implying that lncRNAs are good candidate biomarkers and therapeutic targets for diseases. Intriguingly, the expression of some lncRNAs is deregulated in cancers, and these lncRNAs exert oncogenic or tumor-suppressive functions via various mechanisms, such as regulating the transcription or translation of target genes and modulating signal transduction [9,10]. Furthermore, some lncRNAs are involved in breast cancer progression via controlling some processes of breast cancer pathophysiologies, such as invasion and metastasis, and drug resistance (reviewed in [11]). Thus, lncRNAs may be promising biomarkers and therapeutic targets of cancers, including breast cancer. As mentioned above, endocrine therapy resistance is one of the major therapeutic problems for breast cancer. Intriguingly, some lncRNAs regulate the endocrine therapy resistance of breast cancer, and may be key factors for the treatment of breast cancer with endocrine therapy resistance. In the following sections, we introduce the studies of lncRNAs using models of breast cancer cells that are sensitive or resistant to drugs used for endocrine therapy (e.g., tamoxifen as the SERM, ICI182,780 as the SERD, and anastrozole as the aromatase inhibitor), models of breast cancer cells under LTED conditions, and clinical specimens of breast tumors, and intensely describe the functions and mechanisms of lncRNAs in the endocrine therapy resistance of breast cancer, as revealed by these studies.

## 4. LncRNAs Involved in the Endocrine Therapy Resistance of Breast Cancer

**Figure 1:**
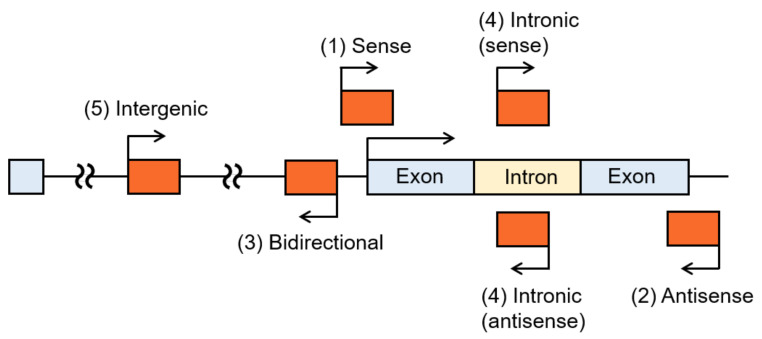
Classification of long noncoding RNAs (lncRNAs). Based on the positions of their loci on the genome, lncRNAs are classified into one or more of five categories: (A) sense, (B) antisense, (C) bidirectional, (D) intronic, and (E) intergenic.

LncRNAs can be classified into one or more of five categories: (A) sense lncRNAs that overlap the neighboring protein-coding gene on the same strand; (B) antisense lncRNAs that overlap the neighboring protein-coding gene on the opposite strand; (C) bidirectional lncRNAs, which are transcribed from the opposite strand within 1 kb from the nearest protein-coding gene; (D) intronic lncRNAs that are derived wholly from intronic regions of protein-coding genes; or (E) intergenic lncRNAs, or long intergenic noncoding RNAs (lincRNAs) that are transcribed from the genomic interval between two genes (Figure 1) [31]. For example, among the lncRNAs related to the endocrine therapy of breast cancer, *HOX transcript antisense RNA* (*HOTAIR*) is classified as an antisense lncRNA, while *urothelial cancer associated 1* (*UCA1*) is a lincRNA. In addition, several endocrine therapy resistance-related lncRNAs belong to multiple categories. In the following section, we introduce lncRNAs involved in the endocrine therapy resistance of breast cancer based on these categories.

### 4.1. Antisense LncRNAs

**Figure 2:**
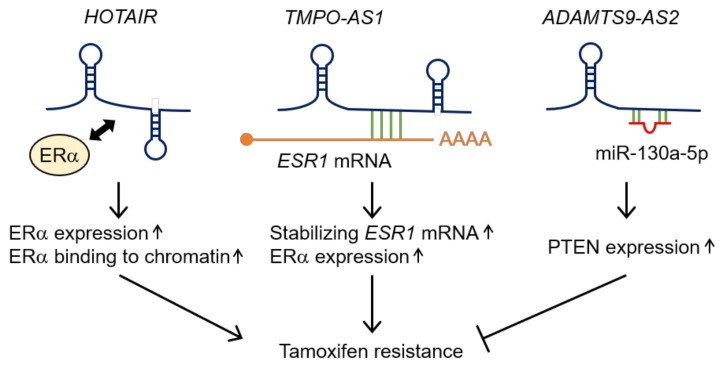
Schematic representation of the functions of *HOTAIR*, *TMPO-AS1*, and *ADAMTS9-AS2* in the tamoxifen resistance of breast cancer. *HOTAIR* enhances tamoxifen resistance by regulating the expression and activity of ERα. *TMPO-AS1* binds and stabilizes *ESR1* mRNA to enhance tamoxifen resistance. On the other hand, *ADAMTS9-AS2* downregulates tamoxifen resistance by competing with miR-130a-5p to increase PTEN expression. *HOTAIR*: *HOX transcript antisense RNA*; *TMPO-AS1*: *thymopoietin antisense transcript 1*; *ADAMTS9-AS*2: *ADAM metallopeptidase with thrombospondin type 1 motif 9* (*ADAMTS9*) *antisense RNA 2*; ERα: estrogen receptor α; PTEN: phosphatase and tensin homolog.

The *HOTAIR* lncRNA enhances resistance to tamoxifen [32]. *HOTAIR* is a ~2.2 kb lncRNA, and its gene overlaps the *homeobox C11* (*HOXC11*) gene on the opposite strand [33]. A previous study using an ER-positive breast cancer cell line, MCF7, showed that *HOTAIR* binds to the estrogen receptor α (ERα), and the overexpression of *HOTAIR* enhances ER signaling by upregulating ERα expression levels and promoting the chromatin binding of the ERα, even under hormone-starved conditions. These results suggest that *HOTAIR* activates ligand-independent ER signaling, which may contribute to tamoxifen resistance (Figure 2) [32]. Moreover, *HOTAIR* has been demonstrated to promote breast cancer progression via transcriptional regulation. *HOTAIR* binds to the polycomb repressive complex 2 (PRC2) with its 5′ side, and regulates the PRC2-mediated trimethylation of H3K27 *in trans* at the *HOXD* locus on chromosome 2, which leads to transcriptional repression of the *HOXD* locus [33]. Furthermore, *HOTAIR* induces selective re-targeting of PRC2 and trimethylated H3K27 genome-wide, thus promoting the invasion of breast cancer cells [34]. Consistently, high expression of *HOTAIR* is associated with a short duration of metastasis-free and overall survival in patients with breast cancer [34,35]. Moreover, the 3′ side of *HOTAIR* binds to corepressor for element-1-silencing transcription factor (CoREST)/repressor element-1 silencing transcription factor (REST) complexes, including lysine-specific demethylase 1 (LSD1), which mediates the demethylation of dimethylated H3K4 (H3K4me2). *HOTAIR* can bind simultaneously to PRC2 and the LSD1/CoREST/REST complexes, to coordinate the targeting of these complexes to hundreds of genes for coupled H3K27 methylation and H3K4 demethylation [36]. Furthermore, the *HOTAIR*–LSD1 complex is involved in transcriptional activation mediated by c-Myc. In addition, the hepatitis B X-interacting protein (HBXIP) binds directly to c-Myc on target genes and recruits LSD1 via interaction with *HOTAIR*, which enhances the transcription of c-Myc target genes, possibly through the LSD1-mediated demethylation of H3K4me2 [37]. *HOTAIR*, HBXIP, and LSD1 promote breast cancer proliferation, highlighting the function of *HOTAIR* as a critical effector of c-Myc in cooperation with HBXIP and LSD1 [37]. Thus, *HOTAIR* plays important roles in the epigenetic regulation of gene expression. However, a recent study has proposed that PRC2 is dispensable for the *HOTAIR*-mediated trimethylation of H3K27 and gene silencing [38], suggesting that further studies are necessary for elucidating the precise mechanisms by which *HOTAIR* epigenetically controls gene expression. In addition, *HOTAIR* acts as a competing endogenous RNA (ceRNA) that specifically sponges the target miRNAs and inhibits their activities. For example, *HOTAIR* is a ceRNA for miR-206 and increases the expression of a miR-206 target gene, the *Bcl-w*/*Bcl-2 like protein 2* (*BCL2L2*) gene, thus promoting breast cancer proliferation [39]. Moreover, *HOTAIR* functions as a ceRNA for miR-20a-5p and upregulates a miR-20a-5p target, the *high mobility group AT-hook 2* (*HMGA2*) gene, which enhances the proliferation, survival, migration, and invasion of breast cancer cells, and the growth of breast tumors [40]. Thus, *HOTAIR* controls breast cancer progression via multiple pathways of regulation of gene expression.

*Thymopoietin antisense transcript 1* (*TMPO-AS1*) is an lncRNA that was demonstrated recently to enhance tamoxifen resistance in breast cancer [41]. *TMPO-AS1* is a ~3.2 kb lncRNA, and its gene overlaps the *thymopoietin* (*TMPO*) gene on the opposite strand. High expression of *TMPO-AS1* is associated with short distant-metastasis-free and overall survival in patients with breast cancer [41]. In addition, the upregulation of *TMPO-AS1* is observed in MCF7-derived, tamoxifen-resistant cells (OHTR) and MCF7-derived LTED cells, which is associated with poor relapse-free survival in patients with breast cancer treated with tamoxifen [41]. *TMPO-AS1* is induced by estrogen in MCF7 cells and another ER-positive breast cancer cell line, T47D. The purification experiments of *TMPO-AS1* from these cell lines by using its antisense oligonucleotide probes suggest that *TMPO-AS1* binds to the 3′ untranslated region (UTR) of the *estrogen receptor 1* (*ESR1*) mRNA, which encodes the ERα protein, through an RNA–RNA interaction. Moreover, this RNA–RNA interaction results in the stabilization of the *ESR1* mRNA. Thus, *TMPO-AS1* upregulates *ESR1* expression and ER signaling pathways, contributing to cell proliferation and tamoxifen resistance (Figure 2) [41]. Therefore, *TMPO-AS1* is a promising biomarker and therapeutic target for ER-positive breast cancer. In other cancers, it has been suggested that *TMPO-AS1* interacts with RNAs other than the *ESR1* mRNA and promotes disease progression [42,43,44]. For example, in cervical cancer, *TMPO-AS1* functions as a ceRNA via the sponging of miR-577 and upregulates a miR-577 target, *RAB14*, to promote cell proliferation, survival, and migration [42]. In osteosarcoma, *TMPO-AS1* increases the expression of *WNT7B* by sponging miR-199a-5p, which promotes cell proliferation and survival [43]. In addition, *TMPO-AS1* stabilizes the *TMPO* mRNA to promote the proliferation, survival, migration, and invasion of non-small cell lung cancer (NSCLC) cells. Mechanistically, *TMPO-AS1* may stabilize the *TMPO* mRNA through their interaction [44]. Thus, *TMPO-AS1* exerts oncogenic effects in various cancers by forming RNA duplexes with some target RNAs.

Conversely, the *ADAM metallopeptidase with thrombospondin type 1 motif 9* (*ADAMTS9*) *antisense RNA 2* (*ADAMTS9-AS2*) lncRNA decreases tamoxifen resistance [45]. *ADAMTS9-AS2* is an antisense transcript of the tumor-suppressor *ADAMTS9* gene. It has been suggested that low expression of *ADAMTS9-AS2* is associated with poor prognosis in patients with several types of cancers, such as lung, colorectal, and gastric cancers, while high *ADAMTS9-AS2* expression is associated with poor prognosis in patients with some cancers, such as bladder cancer and salivary adenoid cystic carcinoma [46,47,48,49,50]. *ADAMTS9-AS2* is downregulated in tamoxifen-resistant cells derived from MCF7, and downregulation of *ADAMTS9-AS2* is also observed in breast cancer tissues, especially in breast tumors with grade III–IV or a tumor size larger than 2 cm [45]. From the knockdown or overexpression experiments of *ADAMTS9-AS2* in an MCF7-derived tamoxifen-resistant cell line, it is shown that *ADAMTS9-AS2* acts as a ceRNA by sponging miR-130a-5p to promote the expression of a miR-130a-5p target gene, *phosphatase and tensin homolog* (*PTEN*), which is a well-known tumor-suppressor and enhances tamoxifen sensitivity (Figure 2) [45].

### 4.2. LincRNAs

**Figure 3:**
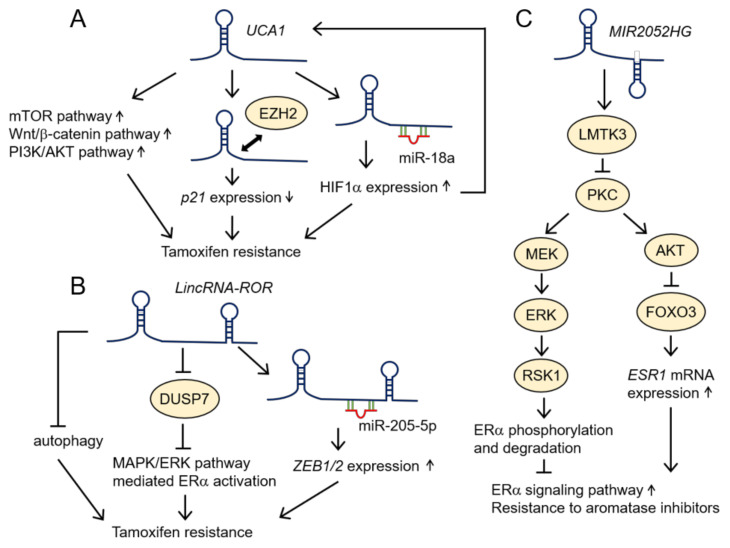
Schematic representation of the functions of *UCA1*, *lincRNA-ROR*, and *lncRNA MIR2052HG* in the tamoxifen resistance of breast cancer. (**A**) *UCA1* promotes the tamoxifen resistance by several mechanisms. *UCA1* activates mTOR, Wnt/β-catenin, and PI3K/AKT signaling pathways to enhance tamoxifen resistance. In addition, *UCA1* binds to EZH2 and epigenetically suppresses *p21* expression. Moreover, *UCA1* sponges miR-18 to upregulate HIF1α expression. Since HIF1α induces *UCA1* expression, *UCA1* and HIF1α form a feedback regulatory loop to strengthen tamoxifen resistance. (**B**) *LincRNA-ROR* enhances tamoxifen resistance by inhibiting autophagy. Moreover, *lincRNA-ROR* induces the degradation of an ERK-specific phosphatase, DUSP7, resulting in ERα activation mediated by the MAPK/ERK signaling pathway. *LincRNA-ROR* also acts as a competing endogenous RNA (ceRNA), which sponges miR-205-5p to upregulate the expression of EMT-related genes *ZEB1/2* and contributes to tamoxifen resistance. (**C**) *MIR2052HG* increases the expression of LMTK3. LMTK3 suppresses the activity of PKC, which increases the expression of *ESR1* mRNA and ERα protein through the inactivation of AKT and MEK/ERK/RSK1 signaling pathway, respectively. *UCA1*: *urothelial cancer associated 1*; *lincRNA-ROR*: *large intergenic noncoding RNA-regulator of reprogramming*; *MIR2052HG*: *miR2052 host gene*; mTOR: mammalian target of rapamycin; PI3K: phosphoinositide 3-kinase; EZH2: enhancer of zeste homolog 2; HIF1α: hypoxia-inducible factor 1α; DUSP7: dual specificity phosphatase 7; MAPK/ERK: mitogen-activated protein kinase (MAPK)/extracellular signal-regulated kinase (ERK); *ZEB1/2*: *zinc finger E-box binding homeobox 1/2*; LMTK3: lemur tyrosine kinase 3; PKC: protein kinase C; *ESR1*: *estrogen receptor 1*; MEK: MAPK/ERK kinase; RSK1: ribosomal S6 kinase 1.

*UCA1* is a lincRNA that was originally identified as a transcript that is upregulated in bladder transitional cell carcinoma [51]. *UCA1* is downregulated in breast cancer and promotes disease progression [52]. From *UCA1* knockdown and overexpression experiments in breast cancer cells, such as MCF7, T47D, and tamoxifen-resistant cells derived from these cells, it has been suggested that *UCA1* enhances tamoxifen resistance by activating the mammalian target of rapamycin (mTOR), Wnt/β-catenin, and PI3K/AKT signaling pathways (Figure 3A) [53,54,55]. Moreover, *UCA1* is shown to interact with the enhancer of zeste homolog 2 (EZH2), which is a catalytic subunit of the PRC2, and suppress the expression of cell-cycle regulator *p21*, by promoting the trimethylation of H3K27 on the *p21* promoter, thus contributing to tamoxifen resistance (Figure 3A) [55]. In addition, from a previous study using breast cancer cell lines, such as MCF7 and BT474, it is shown that *UCA1* acts as a ceRNA by sponging miR-18a to upregulate a target of miR-18a, the *hypoxia-inducible factor 1α* (*HIF1α*). As HIF1α activates the transcription of *UCA1*, *UCA1* and HIF1α form a feedback regulatory loop that strengthens tamoxifen resistance (Figure 3A) [56]. Intriguingly, it has been reported that *UCA1* is secreted from an MCF7-derived, tamoxifen-resistant cell line, LCC2, by exosomes, and that exosome-mediated *UCA1* transfer enhances the tamoxifen resistance of MCF7 cells [57].

Another lincRNA, *breast cancer anti-estrogen resistance 4* (*BCAR4*), was identified in a functional screening of genes regulating the tamoxifen resistance of an ER-positive breast cancer cell line, ZR-75-1 [58]. Further studies suggest that the *BCAR4*-mediated tamoxifen resistance of ZR-75-1 depends on HER2/ErbB2, ErbB3, and ErbB4, but not ERα, and that *BCAR4* overexpression enhances the resistance of MCF7 to antiestrogen ICI182,780 in a HER2/ErbB2-, ErbB3-, and ErbB4-dependent manner. [59,60,61]. *BCAR4* interacts with several proteins, such as glioma-associated oncogene family zinc finger 2 (GLI2), smad nuclear interacting protein 1 (SNIP1), and phosphatase 1 nuclear targeting subunit (PNUTS), and regulates C–C motif chemokine ligand 21 (CCL21)-stimulated noncanonical hedgehog signaling pathway [62]. Although this activity of *BCAR4* contributes to breast cancer metastasis [62], whether this mechanism is involved in the resistance to tamoxifen and ICI182,780 remains unknown.

*Metastasis-associated lung adenocarcinoma transcript 1* (*MALAT1*) is a lincRNA that has been suggested to be involved in the tamoxifen resistance of breast cancer [63]. *MALAT1* was initially reported as an lncRNA that is highly expressed in stage I NSCLC tumors that subsequently metastasize, and high expression of *MALAT1* is associated with short overall survival in patients with NSCLC [64]. Moreover, dysregulation of *MALAT1* expression has been indicated in various cancers, including breast cancer [65]. *MALAT1* is an ~8.7 kb lincRNA, and its gene is located on human chromosome 11q13.1. The *MALAT1* primary transcript contains a tRNA-like structure at the 3′ end [66]. RNase P and RNase Z, which are endonucleases that cleave the 5′ or 3′ side of a tRNA precursor [67,68], cleave both sides of this tRNA-like structure, resulting in the 3′-end maturation of *MALAT1* [66]. The excised tRNA-like RNA (*MALAT1*-associated small cytoplasmic RNA (mascRNA) is added with CCA trinucleotides at the 3′ end and exported to the cytoplasm [66].

Although the function of mascRNA is not well understood, it is suggested that mascRNA is an immune regulator in monocytes that is involved in innate immunity in cardiomyocytes [69]. Moreover, the 3′ end of mature *MALAT1* contains two U-rich sequences and the associated A-rich sequences, and these sequences form a triple-helical structure that enhances the stability of *MALAT1* [70,71]. *MALAT1* is localized in the nucleus, especially in nuclear bodies, which are termed nuclear speckles or SC35 domain and are enriched for splicing factors [72]. Regarding tamoxifen resistance, high expression of *MALAT1* is associated with a short recurrence-free survival in patients with ER-positive breast cancer treated with tamoxifen [63]. In addition, high *MALAT1* expression is associated with poor recurrence-free survival in patients with ER-negative breast cancer, indicating the importance of the ER-independent functions of *MALAT1* [63]. The roles of *MALAT1* in breast cancer are complicated, because both oncogenic and tumor-suppressive roles of *MALAT1* in breast cancer have been reported. For example, *MALAT1* acts as a ceRNA for some miRNAs, such as miR-124, miR-1, miR-129-5p, miR-204, and miR-339-5p, thus promoting breast cancer progression [73,74,75,76,77]. In contrast, *MALAT1* functions as a ceRNA for miR-20a to inhibit the growth and metastasis of breast cancer [78]. Moreover, *MALAT1* regulates transcriptional and posttranscriptional events in ways other than sponging miRNAs. For instance, *MALAT1* interacts with the promoter of the *eukaryotic translation elongation factor 1 alpha 1* (*EEF1A1*) gene and upregulates *EEF1A1* expression by enhancing the trimethylation of H3K4, which promotes the proliferation and invasion of breast cancer [79]. In addition, *MALAT1* forms a complex with the serine/arginine-rich splicing factor 1 (SRSF1), the inhibitor of the DNA binding 4, HLH protein (ID4), and mutant p53, and regulates the alternative splicing of the *vascular endothelial growth factor A* (*VEGFA*) mRNA precursor (pre-mRNA), which increases the angiogenic potential of breast cancer cells [80]. Conversely, *MALAT1* binds to an RNA-binding protein, Hu antigen R (HuR), and interacts with the *CD133* gene to downregulate *CD133*, thus suppressing the epithelial-to-mesenchymal transition (EMT) and migration activity of breast cancer cells [81]. Although the mechanisms via which *MALAT1* exerts both oncogenic and tumor-suppressive functions are not well understood, its functions may depend on context, such as cell type and environment. Furthermore, the dual roles of *MALAT1* in cancer progression have been suggested by studies using *Malat1* knockout (KO) mice [82,83]. Arun et al. reported that *Malat1* KO suppresses the lung metastasis of mammary tumors generated in mouse mammary tumor virus (MMTV)-polyomavirus middle T antigen (PyMT) mice [82]. Inversely, Kim et al. later demonstrated that *Malat1* KO enhances the dissemination and lung metastasis of mammary tumors in MMTV-PyMT mice, and that this phenotype can be rescued by the transgenic expression of *Malat1* from the *ROSA26* locus [83]. Although it is not clear why there is a discrepancy between those results, it may be partly attributed to differences in the methodology for *Malat1* KO. In the former study, *Malat1* KO was accomplished by deletion of a ~3 kb genomic region containing the 5′ end of the *Malat1* gene and its promoter using Cre-mediated recombination technology, while in the latter study, *Malat1* was depleted by inserting the *lacZ* gene and polyadenylation sequences 69 bp downstream of the transcriptional start site of *Malat1*. These genomic rearrangements in *Malat1* KO mice might affect the chromosomal conformation and some nuclear events of gene expression differently, resulting in differential phenotypes. Based on their findings, the manner in which the expression of lncRNAs is suppressed may be important for elucidating lncRNA functions.

The *large intergenic noncoding RNA-regulator of reprogramming* (*lincRNA-ROR*) also upregulates tamoxifen resistance [84,85,86]. *LincRNA-ROR* was originally identified as an lncRNA that is upregulated in induced pluripotent stem cells (iPSCs) compared with embryonic stem cells (ESCs), and has been shown to modulate reprogramming [87]. *LincRNA-ROR* promotes the proliferation and invasion of MCF7 and a TNBC cell line, MDA-MB-231, by regulating the TGF-β signaling pathway, and high expression of *lincRNA-ROR* is associated with a poor prognosis in patients with breast cancer [88]. Regarding the mechanisms by which *lincRNA-ROR* regulates tamoxifen resistance, *lincRNA-ROR* knockdown experiments in BT474 suggest that *lincRNA-ROR* enhances tamoxifen resistance by inhibiting autophagy (Figure 3B) [85]. In addition, a previous study using *lincRNA-ROR-KO* MCF7 cells suggests that *lincRNA-ROR* promotes the degradation of an extracellular signal-regulated kinase (ERK)-specific phosphatase—the dual specificity phosphatase 7 (DUSP7)—under estrogen-deprived conditions, which results in the ligand-independent activation of ERα mediated by the mitogen-activated protein kinase (MAPK)/ERK signaling pathway. As a result, *lincRNA-ROR* promotes estrogen-independent growth and tamoxifen resistance in breast cancer (Figure 3B) [86]. Moreover, from a previous study using an MCF7-derived tamoxifen-resistant cell line, *lincRNA-ROR* is suggested to act as a ceRNA by sponging miR-205-5p to upregulate the miR-205-5p target *zinc finger E-box binding homeobox 1/2* (*ZEB1/2*), thus enhancing EMT and tamoxifen resistance (Figure 3B) [84]. In MDA-MB-231, *lincRNA-ROR* acts as a ceRNA for another miRNA, miR-145, to upregulate its targets, i.e., *ADP ribosylation factor 6* (*ARF6*) and *mucin 1*, which control the subcellular localization of E-cadherin and the metastasis of TNBC [89,90]. In addition to these findings, the genotypes of the rs4801078 SNP in lincRNA-ROR are associated with the risk of breast cancer [91], suggesting that lincRNA-ROR is both a promising biomarker and a therapeutic target of breast cancer.

Furthermore, the lincRNA termed *lncRNA in non-homologous end joining* (*NHEJ*) *pathway 1* (*LINP1*) enhances tamoxifen resistance [92]. *LINP1* was initially identified as an lncRNA that is highly expressed in TNBC. In TNBC, *LINP1* forms a complex with Ku autoantigen, 80kDa (Ku80) and the DNA-dependent protein kinase catalytic subunit (DNA-PKcs) and activates the NHEJ pathway, which repairs double-stranded breaks in DNA [93]. Consistent with these findings, *LINP1* enhances the resistance to radiation and chemotherapeutic drugs that cause DNA damage, such as 5-fluorouracil and doxorubicin [93,94]. *LINP1* also promotes the proliferation of ER-positive MCF7 breast cancer cells [94]. In addition, *LINP1* expression is negatively regulated by estrogen, and is upregulated in ER-positive breast cancer cell lines, MCF7 and T47D, under estrogen-deprived or tamoxifen-treated conditions, as well as in tamoxifen-resistant breast cancer cells derived from these cell lines [92]. From the knockdown and overexpression experiments of *LINP1* in MCF7 and T47D, it is suggested that *LINP1* inhibits the ER signaling pathway by downregulating ERα, which may be involved in tamoxifen resistance [92].

Recently, it has been reported that the *cytoskeleton regulator* (*CYTOR*)/*LINC00152* lincRNA is involved in tamoxifen resistance [95]. *CYTOR* promotes the proliferation and migration of breast cancer cells, and high expression of *CYTOR* is associated with relapse-free survival in patients with breast cancer. *CYTOR* regulates the epidermal growth factor receptor and mTOR signaling pathways and control the organization of the filamentous actin cytoskeleton [96]. Moreover, *CYTOR* is upregulated in tamoxifen-resistant breast cancer cell lines derived from MCF7, and *CYTOR* functions as a ceRNA by sponging miR-125a-5p, and upregulates a target of miR-125a-5p, the *serum response factor* (*SRF*), which promotes the tamoxifen resistance and cell proliferation [95]. Consistent with these data, *CYTOR* expression is higher in breast tumors from tamoxifen-resistant patients [95]. In addition, *CYTOR* is associated with poor prognosis in patients with TNBC, and induces the ubiquitination-mediated degradation of PTEN in TNBC [97].

Although there few studies have addressed lncRNAs that regulate the resistance to aromatase inhibitors, there is some evidence of this phenomenon. The *lncRNA MIR2052HG* lincRNA enhances the resistance to aromatase inhibitors [98,99]. *LncRNA MIR2052HG* is a ~2 kb lncRNA, and its gene is located on human chromosome 8q21.11–q21.13. This lncRNA upregulates the expression of ERα. From the knockdown experiments of *MIR2052HG* in ER-positive CAMA-1 cells, MCF7 cells stably transfected with the *cytochrome P450 family 19 subfamily A member 1* (*CYP19A1*) gene that is an aromatase inhibitor target, and MCF7-derived cells resistant to an aromatase inhibitor anastrozole, it has been shown that *MIR2052HG* increases the expression level of the lemur tyrosine kinase 3 (LMTK3), which in turn regulates the expression of both *ESR1* mRNA and ERα protein, and contributes to the resistance to anastrozole (Figure 3C) [99,100]. For regulating the *ESR1* mRNA, LMTK3 decreases the activity of protein kinase C (PKC), which suppresses Ser 473 phosphorylation and the activation of AKT mediated by PKC. As AKT phosphorylates and induces the proteasome-mediated degradation of forkhead box O3 (FOXO3), a transcription factor that controls *ESR1* expression, the *MIR2052HG*/LMTK3/PKC/AKT axis stabilizes FOXO3, thus upregulating *ESR1* transcription [99,100]. Conversely, LMKT3 suppresses the activity of the mitogen-activated protein kinase (MAPK)/extracellular signal-regulated kinase (ERK) kinase (MEK)/ERK/ribosomal S6 kinase 1 (RSK1) signaling pathway through the downregulation of PKC activity, which results in a decrease in the Ser 167 phosphorylation level of ERα as well as its stabilization (Figure 3C) [99,100]. In addition, single-nucleotide polymorphisms (SNPs) located near or within the *MIR2052HG* gene locus are associated with the recurrence of breast cancer in patients treated with aromatase inhibitors as adjuvant therapy, suggesting that these SNPs in *MIR2052HG* are promising biomarkers that can be used to identify patients in whom aromatase inhibitors would be an appropriate therapy [98].

Moreover, a recent study indicated that the lncRNA *LINC00309* is associated with poor disease-free survival in patients with breast cancer treated with endocrine therapy using aromatase inhibitors, which suggests that *LINC00309* plays important roles in the acquisition of resistance to these therapeutic agents [101].

### 4.3. LncRNAs Belonging to Multiple Categories

**Figure 4:**
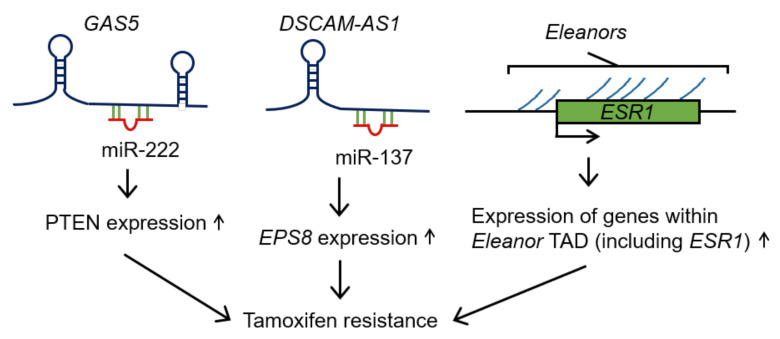
Schematic representation of the functions of *GAS5*, *DSCAM-AS1*, and *Eleanors* in the tamoxifen resistance of breast cancer. *GAS5* sponges miR-222 and upregulates PTEN expression to enhance tamoxifen resistance. *DSCAM-AS1* also sponges miR-137 to increase *EPS8*, which contributes to tamoxifen resistance. On the other hand, *Eleanors* promotes tamoxifen resistance by upregulating *ESR1* expression. *GAS5*: *growth-arrest specific 5*; *DSCAM-AS1*: *Down syndrome cell adhesion molecule antisense RNA 1*; *Eleanors*: *ESR1 locus enhancing and activating noncoding RNAs;* PTEN: phosphatase and tensin homolog; *EPS8*: *epidermal growth factor receptor pathway substrate 8*.

The *growth-arrest specific 5* (*GAS5*) lncRNA is downregulated in tamoxifen-resistant breast cancer cells, and low *GAS5* expression enhances resistance to tamoxifen [102]. *GAS5* was originally isolated as a gene that is preferentially expressed in growth-arrested NIH3T3 cells [103]. The *GAS5* gene has two alternative promoters, as well as multiple exons and introns. As alternative choices of these exons and alternative promoter usage produce multiple *GAS5* variants, *GAS5* can be defined as an antisense lncRNA overlapping a protein-coding gene, the *zinc finger and BTB domain containing 37* (*ZBTB37*) gene, on the opposite strand or as a bidirectional lncRNA [104]. *GAS5* is a host gene of a class of small　noncoding RNAs termed box C/D small nucleolar RNAs (SNORDs); 10 SNORDs are encoded within the *GAS5* intronic regions [104]. These snoRNAs are transcribed as part of the *GAS5* primary transcript, and are then excised and matured. Regarding endocrine therapy resistance, a previous study using an MCF7-derived tamoxifen-resistant cell line suggests that *GAS5* acts as a ceRNA by sponging miR-222 and upregulates *PTEN*, which is a target of miR-222 and weakens the tamoxifen resistance of breast cancer cells (Figure 4) [102].

In addition to downregulating tamoxifen resistance, *GAS5* exerts tumor-suppressive effects in breast cancer via several pathways. For example, *GAS5* acts as a ceRNA by sponging miR-21 and upregulates the expression of miR-21 targets *programmed cell death 4* (*PCDC4*) and *PTEN*, both of which are tumor-suppressor genes [105]. Moreover, *GAS5* acts as a ceRNA for miR-196a-5p and downregulates the forkhead box O1 (FOXO1)/phosphoinositide 3-kinase (PI3K)/AKT pathway, thus suppressing the invasion of TNBC cells [106]. In addition to its functions as a ceRNA, *GAS5* is involved in transcriptional regulation. *GAS5* suppresses glucocorticoid-induced transcription and sensitizes breast cancer cells to apoptosis [107,108]. Exon 12 of *GAS5* contains a hairpin structure with two sequences similar to the GR target sequence, termed glucocorticoid response element (GRE). This hairpin structure is called the *GAS5* GRE-mimic, and it interacts directly with the DNA-binding domain of GR and suppresses the transcriptional activation of GR target genes, including antiapoptotic genes like *cellular inhibitor of apoptosis 2* (*cIAP2*) and *serum- and glucocorticoid-regulated kinase 1* (*SGK1*), which facilitate stress-inducible apoptosis [107]. In addition to the GR, *GAS5* and the *GAS5* GRE-mimic bind to other 3-keto steroid receptors, such as the mineralocorticoid, progesterone, and androgen receptors, and inhibit their transcriptional activities [107,108]. Interestingly, the *GAS5* GRE-mimic alone can increase apoptosis in breast cancer cells, suggesting that the oligonucleotides of the GRE-mimic may be applicable to breast cancer therapy [108]. Consistent with these findings, *GAS5* is downregulated in breast tumors compared with normal tissues, and low expression of *GAS5* is associated with poor overall survival in patients with breast cancer and TNBC [102,104,106]. Moreover, a recent study showed that an insertion (ins)/deletion (del) polymorphism located within the *GAS5* promoter (rs145204276 AGGCA/–) affects the risk of breast cancer [109]. In that study, the rs145204276 ins/del and del/del genotypes, as well as the del allele, were associated with a reduced risk of breast cancer [109]. As *GAS5* expression is significantly higher in patients with breast cancer carrying the rs145204276 ins/del and del/del genotypes versus the rs145204276 ins/ins genotype carriers, and since the rs145204276 del allele increases the transcription of *GAS5*, this polymorphism may affect the risk of breast cancer by modulating *GAS5* expression levels [109].

The *Down syndrome cell adhesion molecule antisense RNA 1* (*DSCAM-AS1*) is an intronic antisense lncRNA that is transcribed from the opposite strand of the *Down syndrome cell adhesion molecule* (*DSCAM*) gene, and is wholly derived from the intronic region of *DSCAM*. *DSCAM-AS1* promotes tamoxifen resistance [110,111] and is upregulated in breast cancer tissues compared with normal tissues [110,112]. Moreover, *DSCAM-AS1* expression is higher in luminal and HER2-positive breast cancers, and particularly in the luminal B subtype [110,112]. Importantly, previous studies have demonstrated that *DSCAM-AS1* is an ERα target gene [110,112], and is important for cell proliferation and the invasion of MCF7 and T47D cells [110,112,113], as well as for the growth and liver metastasis of T47D cells xenografted into immunodeficient mice [110]. Moreover, *DSCAM-AS1* expression is elevated in tamoxifen-resistant breast cancer tissues, and the knockdown and overexpression experiments of *DSCAM-AS1* in breast cancer cell lines, such as MCF7 and T47D, suggest that *DSCAM-AS1* promotes tamoxifen resistance [110,111]. Consistent with these results, a high expression of *DSCAM-AS1* has been associated with a short disease-free survival for patients with luminal breast cancer and those with luminal breast cancer treated with endocrine therapy [113]. Although the manner in which *DSCAM-AS1* functions in breast cancers remains unclear, the RNA-binding protein heterogeneous nuclear ribonucleoprotein L (hnRNPL) is required for *DSCAM-AS1* activity in MCF7 and T47D cells [110]. *DSCAM-AS1* interacts with hnRNPL via its 3′ region, which contains CACA-rich RNA sequences [110]. Furthermore, a previous study using MCF7-derived, tamoxifen-resistant cells suggests that *DSCAM-AS1* acts as a ceRNA by sponging miR-137, which increases the expression of *epidermal growth factor receptor pathway substrate 8* (*EPS8*), thus contributing to tamoxifen resistance (Figure 4) [111]. In addition, it was reported recently that *DSCAM-AS1* functions as a ceRNA for miR-204-5p in the *breast cancer susceptibility gene 1* (*BRCA1*)-mutated TNBC cell line HCC1937, to promote tumor growth via the upregulation of *ribonucleotide reductase M2* (*RRM2*) [114].

*ESR1 locus enhancing and activating noncoding RNAs* (*Eleanors*) were identified as a group of lncRNAs that are transcribed from inside and around the *ESR1* locus, and could consist of lncRNAs of all categories [115]. Previous studies have shown that *Eleanors* play important roles in ER-positive breast cancer progression under estrogen-deprived conditions. *Eleanors* are specifically expressed in ER-positive breast cancer tissues and MCF7 cells, and are increased in MCF7 cells cultured under LTED conditions [115]. *u-Eleanor* is an *Eleanor* that is transcribed from ~40 kb upstream of the canonical promoter of *ESR1* and upregulates the transcription of the *ESR1* mRNA and other *Eleanors* to promote the proliferation of LTED cells (Figure 4) [115]. A chromatin immunoprecipitation-sequencing (ChIP-seq) analysis showed that the *u-Eleanor* locus in LTED cells is enriched for monomethylated H3K4, rather than trimethylated H3K4, suggesting that the *u-Eleanor* locus functions as an enhancer. Clinically, the upregulation of *u-Eleanor* has been reported to be negatively associated with increasing breastfeeding duration [116]. *u-Eleanor* tends to be upregulated in healthy women without a history of breastfeeding and women with a breastfeeding duration of 1–6 months. Epidemiological studies have demonstrated that breastfeeding experiences play a protective role against breast cancer in women, while a lack or a short duration of breastfeeding increases breast cancer risk [117,118,119]. Therefore, *u-Eleanor* may be used as a biomarker of breast cancer at early stages [116]. Furthermore, a recent study has revealed the function of another *Eleanor* called promoter-associated *Eleanor* (*pa-Eleanor*), which is transcribed from the region proximal to the transcriptional start site of *ESR1* [120]. In the nucleus, chromosomes fold into domains called topologically associating domains (TADs), which exhibit intra-chromatin interactions [121]. The chromosome conformation capture combined with high-throughput sequencing (4C-seq) analysis, reported in a recent study, reveals that a TAD including *Eleanor*-expressing regions (*Eleanor* TAD) resides on human chromosome 6q25.1, and that *Eleanor* TAD contains the *ESR1* gene and three other genes: *coiled-coil domain containing 170* (*CCDC170*), *chromosome 6 open reading frame 211* (*C6orf211*), and *required for meiotic nuclear division 1 homolog* (*RMND1*) [120]. *pa-Eleanor* upregulates genes within *Eleanor* TAD and promotes the proliferation of LTED cells (Figure 4). In addition, *pa-Eleanor* upregulates *u-Eleanor*, whereas *u-Eleanor* does not affect *pa-Eleanor* expression, suggesting that *pa-Eleanor* upregulates the transcription of the *ESR1* mRNA through *u-Eleanor* [120]. Moreover, *pa-Eleanor* enhances an intra-chromosomal interaction between the *ESR1* promoter region and the region near the *FOXO3* locus on human chromosome 6q21 [120]. This chromosomal interaction may affect the expression of genes within *Eleanor* TAD. FOXO3 is a transcription factor that induces apoptosis through the transcriptional regulation of apoptosis-associated genes, and its expression is elevated in LTED cells. The knockdown of *pa-Eleanor* decreases *ESR1* expression levels (but does not affect the expression of FOXO3) and induces the apoptosis of LTED cells. Therefore, *pa-Eleanor* may promote the survival of LTED cells by regulating the balanced expression of *ESR1* and *FOXO3* [120]. Thus, the functions of *u-Eleanor* and *pa-Eleanor* suggest that the regulation of *Eleanor* expression may represent a new treatment strategy for breast cancer adapted to estrogen-deprived conditions. Consistent with this idea, resveratrol and glyceollin I, phytoalexins that are synthesized in plants under stress conditions, decrease the expression of *Eleanors* to induce apoptotic death in LTED cells [122].

In addition to these lncRNAs, recent gene expression analysis in patients with ER-positive breast cancer, who were primarily treated with tamoxifen, identified 11 lncRNAs, belonging to multiple categories (*PINK1-AS*, *RP11-259N19.1*, *KLF3-AS1*, *LINC00339*, *LINC00472*, *RP11-351I21.11*, *KB-1460A1.5*, *PKD1P6-NPIPP1*, *PDCD4-AS1*, *PP14571*, and *RP11-69E11.4*), as prognostic lncRNAs that predict the risk of systemic relapse [123]. *PINK1-AS*, *RP11-259N19.1*, *KLF3-AS1*, *PDCD4-AS1*, *PP14571*, and *RP11-69E11.4* are antisense lncRNAs, while *LINC00339*, *LINC00472*, *RP11-351I21.11*, and *KB-1460A1.5* are lincRNAs. *PKD1P6-NPIPP1* is a read-through transcript derived from two pseudogenes, *polycystin 1, transient receptor potential channel interacting pseudogene 6* (*PKD1P6*) and *nuclear pore complex interacting protein pseudogene 1* (*NPIPP1*), and classified as an intronic antisense lncRNA, because *PKD1P6-NPIPP1* is wholly derived from the opposite strand of the intronic region of the *pyridoxal dependent decarboxylase domain containing 1* (*PDXDC1*) gene. Although the mechanisms by which these 11 lncRNAs are involved in tamoxifen resistance and systemic relapse are unclear, several relapse- or metastasis-related pathways, such as the PI3K/AKT and Wnt signaling pathways, are upregulated in patients with breast cancer who have a high relapse risk predicted by the expression levels of these lncRNAs. Thus, it suggests that these signaling pathways may play important roles in the functions of the 11 prognostic lncRNAs [123].

## 5. Antisense Oligonucleotides (ASOs) in Clinical Use

**Figure 5:**
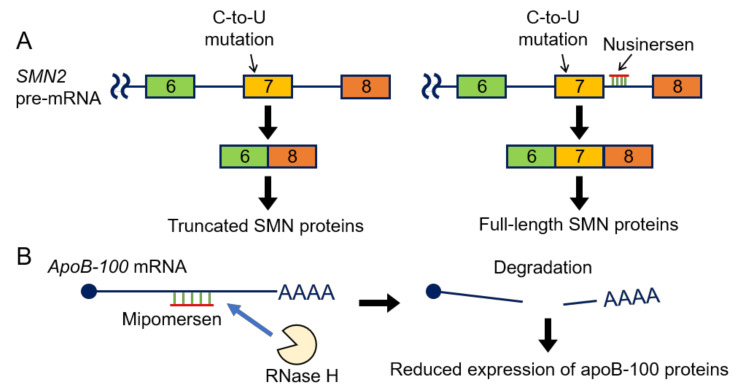
Antisense oligonucleotides (ASOs) in clinical use. (**A**) Nusinersen binds to a splicing regulatory sequence called intronic splicing silencer-N1 (ISS-N1) within intron 7 of SMN2 pre-mRNA, and enhances the inclusion of exon 7, resulting in the production of *SMN2* mRNA coding the full-length SMN protein. (**B**) Mipomersen binds to *ApoB-100* mRNA and causes its degradation, mediated by ribonuclease H (RNaseH). *SMN2*: *survival of motor neuron 2: centromeric*; SMN: survival of motor neuron; *ApoB-100*: *apolipoprotein B-100*.

Considering that lncRNAs play essential roles in endocrine therapy resistance, intervention against lncRNAs may be promising for breast cancer treatment. Antisense oligonucleotides (ASOs) are used for regulating the stability and activity of RNAs. Some chemically modified ASOs targeting transcripts of protein-coding genes have been approved for clinical use by the U.S. Food and Drug Administration (FDA) [124]. For example, a 2′-O-(2-methoxyethyl) (MOE) phosphorothioate (PS) ASO called nusinersen is used for the treatment of spinal muscular atrophy (SMA). [125,126]. SMA is an autosomal-recessive neuromuscular disorder with degeneration of the motor neurons in the anterior horn of the spinal cord, leading to atrophy of the voluntary muscles of the limbs and trunk [125]. SMA is caused by deletions or loss-of-function mutations of *survival of motor neuron 1, telomeric* (*SMN1*) gene and the consequent reduced expression of survival of motor neuron (SMN) proteins from *SMN1* transcripts. Although there is a homologue of *SMN1* gene called *survival of motor neuron 2, centromeric* (*SMN2*), SMN proteins are not efficiently produced from *SMN2* transcripts. The *SMN2* gene has an identical coding sequence but differs from *SMN1* gene by 11 nucleotides. The different sequences between these genes contain a C-to-T mutation on exon 7, which is a synonymous mutation but affects SMN protein expression by promoting the skipping of exon 7. Due to this mutation, 80%–90% of *SMN2* mRNAs lack exon 7 and are translated into truncated SMN proteins, which are rapidly degraded (Figure 5A). Therefore, the *SMN2* gene does not fully compensate for the loss-of-function of the *SMN1* gene [125], and modulating the splicing pattern of *SMN2* pre-mRNA to produce the full-length SMN proteins is one of therapeutic strategies of SMA. Nusinersen is an ASO complementary to a site within intron 7 of the *SMN2* pre-mRNA called intronic splicing silencer-N1 (ISS-N1), which is involved in the skipping of exon 7 and blocks the activity of ISS-N1 to facilitate the inclusion of the exon 7, resulting in the synthesis of the functional, full-length SMN proteins and the rescue of motor neurons (Figure 5A) [124,125]. Another oligonucleotide drug, mipomersen, is used to treat homozygous familial hypercholesterolemia, an autosomal disorder of the lipid metabolism characterized by elevated levels of low-density lipoprotein (LDL) cholesterol [124,127]. Mipomersen targets the transcripts of the *apolipoprotein B* (*APOB*) gene. The middle region of mipomersen shows DNA-like properties and induces the cleavage of these transcripts mediated by ribonuclease H (RNase H), which cleaves RNAs that form heteroduplexes with DNA. As the apoB-100 protein, encoded by *APOB* gene, is a component of LDL cholesterol, mipomersen-mediated downregulation of *APOB* decreases the circulating levels of LDL cholesterol (Figure 5B) [123,126,128]. In addition to ASOs, a small interfering RNA (siRNA)-based drug, patisiran, was recently approved by the FDA [124]. Therefore, the targeting lncRNAs with ASOs and siRNAs may be translated into new therapies for breast cancer.

## 6. Conclusions

In this review, we describe the functions and mechanisms of lncRNAs related to the endocrine therapy resistance of breast cancer (Table 1), and their potential as therapeutic targets. Additionally, LncRNAs may hold promise as biomarkers of breast cancer. Importantly, the quantification of the prostate cancer antigen 3 (*PCA3*) lncRNA in urine samples has been developed as a diagnostic test for prostate cancer [129], suggesting that lncRNAs may be applicable to the analysis of non-invasive liquid biopsies for the diagnosis of cancers, including breast cancer. Thus, lncRNAs are potential key factors in the development of new strategies of breast cancer treatment, and further studies of lncRNAs in the context of breast cancer are required.

## Figures and Tables

**Table 1:** LncRNAs regulating endocrine therapy resistance in breast cancer.

LncRNAs	Effects on Endocrine Therapy Resistance	Molecular Mechanisms Regulating Endocrine Therapy Resistance	Breast Cancer Cells or Tissues Used for Analyzing the Mechanisms of LncRNAs
Antisense lncRNAs			
*HOTAIR*	Up-regulation	Upregulating the activity of ERα under estrogen-starved condition [32]	MCF7 and an MCF7-derived tamoxifen-resistant cell line (Tam^R^ MCF7) [32]
*TMPO-AS1*	Up-regulation	Upregulating *ESR1* mRNA stability [41]	MCF7, T47D, and MCF7-derived tamoxifen-resistant cells (OHTR cells), as well as MCF7 cultured under long-term estrogen-deprivation conditions (LTED cells) [41]
*ADAMTS9-AS2*	Down-regulation	Inhibiting miR-130a-5p activity to increase the expression of *PTEN* [45]	MCF7 and an MCF7-derived tamoxifen-resistant cell line (MCF-7R) [45]
*PINK1-AS*	Down-regulation	Possibly regulating several relapse or metastasis-related pathways, such as PI3K/AKT and Wnt signaling pathways [123]	ER-positive breast tumors from patients who were primarily treated with tamoxifen as the unique endocrine therapy [123]
*RP11-259N19.1*	Up-regulation		
*KLF3-AS1*	Down-regulation		
*PDCD4-AS1*	Down-regulation		
*PP14571*	Up-regulation		
*RP11-69E11.4*	Down-regulation		
LincRNAs			
*UCA1*	Up-regulation	a) Activating mTOR, Wnt/β-catenin, and PI3K/AKT signaling pathways [53,54,55]; b) Promoting EZH2 mediated repression of *p21* [55]; c) Inhibiting miR-18a activity to increase HIF1α expression [56]	MCF7 and MCF7-derived tamoxifen- and ICI182,780-resistant cell lines (LCC2 and LCC9) [53]; MCF7, T47D, and tamoxifen-resistant cells derived from MCF7 and T47D (MCF-7-R and T47D-R) [54]; LCC2 [55], MCF7, BTB474, LCC2, and LCC9 [56]
*BCAR4*	Up-regulation	Upregulating tamoxifen resistance of ZR-75-1 and ICI182,780 resistance of MCF7 in a HER2/ErbB2-, ErbB3-, and ErbB4-dependent manner [58,59,60,61]	ZR-75-1 [58,59,60], MCF7 [61]
*MALAT1*	Up-regulation [62]	Unknown	
*LincRNA-ROR*	Up-regulation	a) Inhibiting autophagy [85]; b) Promoting ligand-independent activation of ERα and estrogen-independent growth [86]; c) Inhibiting miR-205-5p activity to increase the expression of *ZEB1/2* [84]	BT474 [85]
			MCF7 [86]
			MCF7, an MCF7-derived tamoxifen-resistant cell line (MCF7/TR5), and MDA-MB-231 [84]
*LINP1*	Up-regulation	Decreasing ERα expression level [92]	MCF7, T47D, tamoxifen-resistant cells derived from MCF7 and T47D (MCF-7/TAMR and T47D/TAMR) [92]
*CYTOR*	Up-regulation	Inhibiting miR-125a-5p to increase the expression of *SRF* [95]	MCF7-derived tamoxifen-resistant cell lines (MCF7/TAM1 and MCF7/TAM2) [95]
*MIR2052HG*	Up-regulation	Increasing the expression of LMTK3 to upregulate ERα expression [99]	CAMA-1, an MCF7 cell line stably transfected *CYP19A1* (MCF7/AC1), and an anastrozole-resistant MCF7 cell line (MCF7/AnaR) [99]
*LINC00309*	Up-regulation [100]	Unknown	
*LINC00339*	Down-regulation	Possibly regulating several relapse or metastasis-related pathways, such as PI3K/AKT and Wnt signaling pathways [123]	ER-positive breast tumors from patients who were primarily treated with tamoxifen as the unique endocrine therapy [123]
*LINC00472*	Down-regulation		
*RP11-351I21.11*	Down-regulation		
*KB-1460A1.5*	Up-regulation		
Other lncRNAs			
*GAS5*	Down-regulation	Inhibiting miR-222 activity to increase the expression of *PTEN* [102]	MCF-derived, tamoxifen-resistant cell line (MCF-7R) [102]
*DSCAM-AS1*	Up-regulation	Inhibiting miR-137 to increase *EPS8* [111]	MCF7-derived, tamoxifen-resistant cells (TR MCF7) [111]
*Eleanors*	Up-regulation	Increasing the expression of *ESR1* [115.120]	MCF7 and MCF7 cultured under long-term estrogen-deprivation conditions (LTED cells) [115,120]
*PKD1P6-NPIPP1*	Down-regulation	Possibly regulating several relapse or metastasis-related pathways, such as PI3K/AKT and Wnt signaling pathways [123]	ER-positive breast tumors from patients who were primarily treated with tamoxifen as the unique endocrine therapy [123]
